# Molecular Dynamics Simulations of the Structural and Thermodynamic Properties of Poly( *l* ‑lactic acid) in the Presence of Water

**DOI:** 10.1021/acs.macromol.6c00782

**Published:** 2026-06-01

**Authors:** Kalle Tuomi, Terttu I. Hukka, Jurkka Kuusipalo, Mikko Karttunen

**Affiliations:** † Faculty of Engineering and Natural Sciences, Materials Science and Environmental Engineering, Chemistry & Advanced Materials, 201768 Tampere University, P.O. Box 541, FI-33014 Tampere University, Finland; ‡ Faculty of Engineering and Natural Sciences, Materials Science and Environmental Engineering, Paper Converting and Packaging Technology, 201768 Tampere University, P.O. Box 589, FI-33014 Tampere University, Finland; § European Laboratory for Learning and Intelligent Systems (ELLIS) Institute Finland, Maarintie 8, 02150 Espoo, Finland; ∥ Department of Technical Physics, University of Eastern Finland (UEF), P.O. Box 1627, FI-70211 Kuopio, Finland; ⊥ Department of Physics and Astronomy, The University of Western Ontario, 1151 Richmond Street, London, Ontario N6A 3K7, Canada; # Department of Chemistry, 6221 The University of Western Ontario, 1151 Richmond Street, London, Ontario N6A 5B7, Canada; ∇ Department of Physics, 201768 Tampere University, P.O. Box 600, FI-33014 Tampere University, Finland

## Abstract

In this study, we used all-atom molecular dynamics (MD) simulations to investigate the behavior of poly­( *l* -lactic acid) (PLLA) in the presence of 0, 1, 2, 6, and 12 m-% water. We focus on understanding how the contacts between various atoms of adjacent PLLA chains evolve and how these changes influence the system’s volume, density, and mechanical and thermodynamic properties. We observe that as the solvent concentration increases, the number of contacts between the atoms of the adjacent PLLA chains decreases, leading to swelling when water penetrates between the polymer chains. However, a small amount of water is seen to facilitate contacts between the oxygen and hydrogen atoms, and the volume of the system contracts. This variation in interchain contacts correlates with changes in density. The contraction is also reflected in the thermal expansion coefficient, which decreases slightly at 1–2 m-%, but then increases at higher water contents due to swelling. In addition, the isothermal compressibility decreases and the isothermal bulk modulus increases; in other words, the resistance to compression increases as 2–6 m-% water is added. Moreover, the specific heat capacity increases after 6 m-%, by 11% at 12 m-%. Our simulation results show strong agreement with the experimental observations. The insights gained from this study improve our understanding of the molecular interactions between PLLA chains and the mechanistic degradation steps favored in the presence of water. These findings have significant implications for the design of sustainable and biodegradable materials, with applications ranging from food packaging to medical uses.

## Graphical abstract

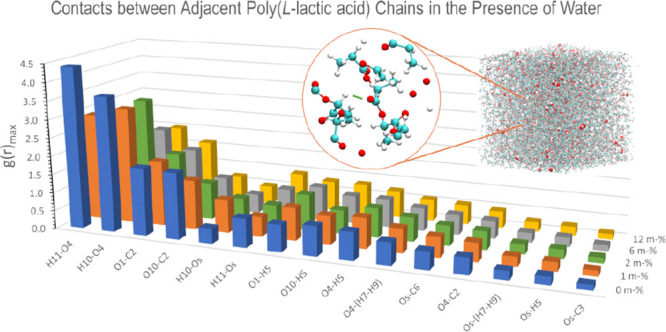

## 1 Introduction

Poly­(lactic acid) (PLA), or polylactide, is a widely used^1,2^ biobased^3^ and biodegradable^4–6^ thermoplastic,^7^ whose production binds carbon dioxide^8^ unlike petrochemical polymers. The microbial fermentation of renewable feedstocks generates lactic acid (LA) monomers.^9,10^ Two enantiomers exist, i.e., (*S*)- and (*R*)- alias *L*- and *D*-stereoisomers,^11,12^ respectively, due to the chiral α-carbon. Aliphatic polyester (i.e., poly­[oxy­(1-methyl-2-oxoethylene)] or poly­(2-hydroxy propanoic acid)), PLA, can be synthesized^13,14^ via a step growth mechanism (polycondensation) from LA monomers or through a combined step and chain growth polymerization, i.e., ring opening of cyclic lactide dimers, producing isotactic or syndiotactic PLA, respectively.^15,16^ The synthesis route,^17^ mutual monomer reactivity, enantiomeric composition,^11,18^ and subsequent processing^19,20^ influence its structural,^17,21^ chemical, physical, thermal, and mechanical properties. When using an enantiomer of *S* type, semicrystalline morphologies^22^ with syndio- ^11,23^ (*trans*-) or isotactic^24^ PLLA chains have been reported. However, the semicrystalline morphology of *c*-PLLA becomes amorphous (*a*-PLLA) and atactic when the polymer is rapidly cooled from the molten state, as occurs, e.g., during injection molding^25^ and paperboard coating.^26^ As a result, the mechanical properties change,^25,27^ the barrier against oxygen^28^ and water^27,29–32^ permeation decreases, and even 1% of water inhibits the cold-crystallization observed in *c*-PLLA.^31^

PLA degrades^33,34^ chemically, by abiotic hydrolysis, to biocompatible lactic acid under favorable aqueous conditions. Biocompatibility fits PLA for biomedical and biotechnological applications, but it is also used in packaging^35–40^ and long-life^3,41^ consumer products. Lactic acid can further biodegrade to H_2_O, CO_2_, CH_4_, and biomass under enzymatic and microbial,^42^ i.e., biotic, action in even a slightly humid environment.^43^ This makes PLA suitable for environmentally friendly goods, that is, disposable and compostable^34^ commodities and biodegradable printed electronics.^44^ Considering the wide range of applications and its increasing importance as an engineering polymer, it is clear that understanding the chemistry and interactions between water and PLA is very important. However, intermolecular interactions have not yet been fully addressed. To achieve this, a detailed understanding of the interactions between PLA chains is required first in the absence and presence of water, before the role of water in disrupting and modifying these interactions can be understood at the atomic level. That is the focus of this article.

Blomqvist et al.^45^ were the first to use MD simulations to study water uptake of amorphous poly­( *l* -lactic acid) (PLLA) and poly­( *l* -lactide-*co*- *d* -lactide) (PLLA-*co*-PDLA). They used a model that contained 10 H_2_O molecules (approximately 1 m-%) and five polymer chains consisting of 50 constitutional repeating units (CRUs) each (approximately 3600 g/mol). No significant differences in the packing of the pure amorphous structures were reported. However, they concluded that the number of hydrophobic CH_3_ groups was the main factor controlling the probability of water uptake. However, the water concentration was small and the model was built in such a way that only the interactions with carbonyl groups were predicted. In a related experimental study, Hofmann et al.^46^ conducted a comparative modeling study on racemic poly­( *d* , *l* -lactide) (PDLLA) and poly­(glycolic acid) (PGA). They concluded that as a result of the bulky methyl groups, PLLA and PDLLA have larger free volumes, which increase more with water content (swelling) than those of PGA. However, PLA tacticity did not significantly affect free volume, water solubility, or water uptake. Hofmann et al. concluded that water solubility controls water uptake and increases when the number of more polar groups increases, that is, polarity, solubility, and water uptake are greater in PGA than in PLLA.^46^ Li et al.^47^ simulated the interactions of water with PLA using MD and reported changes in the polymer conformations. Despite studies, information on the exact PLA structures at the atomic level in moist environments and tacticity of the chains is unclear.

It has been determined experimentally that the less crystalline and amorphous PLLA samples (*a*-PLLA) permeate^48^ and transmit water^49^ faster, and that degradation proceeds more rapidly in the center than at the surface,^50,51^ starting in the amorphous regions.^52^ Cairncross et al. hypothesized that moisture sorption is controlled by hydrophilic end groups in PLA.^53,54^ In PDLLA, water causes swelling and “plasticization” of the polymer structure. Water reduces the glass transition temperature (*T*_g_) of PDLLA,^55,56^ similarly to other polymers,^57^ and hence decreases the specific and free volumes^58,59^ before any bond cleavage occurs. Semicrystalline PLA (*c*-PLA) absorbs water even at relative humidities of 100% due to degradation products, but when immersed, the degradation products transfer to the surrounding aqueous medium and water sorption ceases.^60^

In this work, we used all-atom MD simulations to investigate how the structural, chemical, mechanical, and thermodynamic properties of *a*-PLLA depend on the concentration of water. The systems consist of varying concentrations of water (pure PLLA and PLLA with 1, 2, 6, and 12 m-% of water) and 25 chains with 150 CRUs of PLLA, allowing us to study these properties and contacts between adjacent polymer chains in bulk structures. Our models enable addressing of the atomic contacts between the PLLA chains and those induced by the chain end groups.

## 2 Models and Methods

In this work, the *a*-PLLA model (with 100% *L* isomers) (Figure 1) of Glova et al.^61^ with 150 CRUs and their General AMBER Force Field^62^ (GAFF) parametrization was employed. The PLLA system had 25 chains and the TIP3P^63^ model was used for water. All simulations were performed using the Gromacs 2020.4 package.^64^ Systems with four different percentages of water (approximately 1, 2, 6, and 12 m-%) were prepared. The molar mass of the PLLA model was approximately 11 kg/mol, which is well above the critical molar mass of 9 kg/mol^65,66^ needed for stable entanglements. As stated by Glova et al.^61^ these PLLA chains are long enough to give reliable data in MD simulations, although the molar mass is lower than that of the PLLA used in experiments. This PLLA model has also been used successfully in previous studies of composites.^67,68^ Experimentally, the *T*_g_ of dry PDLLA is 315 K^55^ and decreases by 10 to 305 K after the addition of 1.2 m-% water. Adding 2 m-% is expected to further decrease *T*_g_. In addition, shorter polymer chains and a more amorphous morphology are known to lower *T*_g_. The *T*_g_ of the dry PLLA was 330–340 K^69^ for a simulation system using the same force field as used in this work.

**Figure 1:**
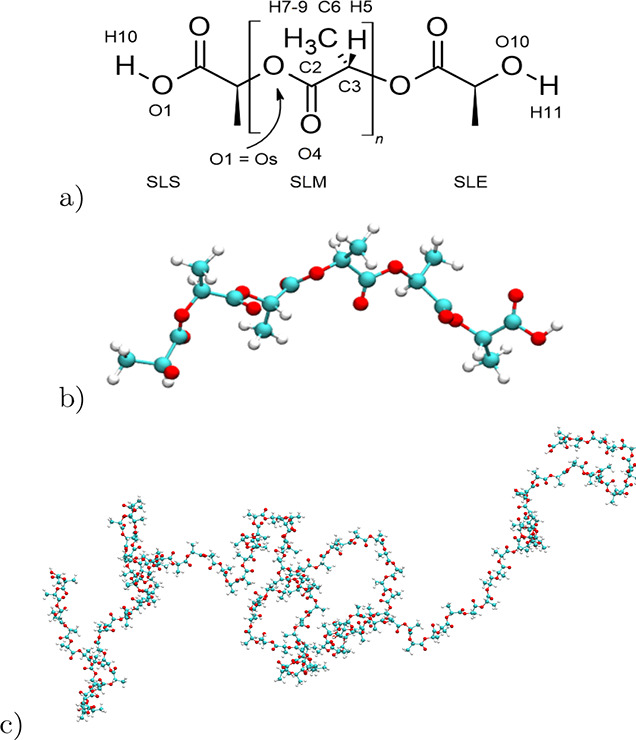
(a) Constitutional repeating unit (CRU) of a poly­( *l* -lactide) and labeling of the atom (Single Lactide Start SLS = terminal CRU with a carboxyl group, Mid SLM = chain mid CRUs, End SLE = terminal CRU with an hydroxy group); (b) portion of a syndiotactic PLLA chain (cyan = carbon, red = oxygen, white = hydrogen), and (c) a starting structure of a single 150 CRUs long PLLA chain with no water was present. The PLLA systems for the MD simulations consisted of 25 PLLA chains.

### 2.1 System Preparation

First, a pure PLLA system was created to simulate an *a*-PLLA matrix. As the density of the initial box was low, the system was compressed to adjust the density closer to the experimental values.^35^ To speed up equilibration, the temperature was first set at 550 K and the system was compressed with a pressure of 50 bar in the NpT ensemble using the Berendsen barostat and thermostat;^70^ although 550 K is much higher than the boiling point of water, the PLLA chain matrix kept the system stable, as molecular decomposition is not possible in classical MD simulations.

In the next step, the pressure was reduced to 1 bar in steps of 10 bar with 2 ns long simulations at 550 K temperature in the NpT ensemble. At 1 bar, the pure PLLA system was then relaxed using the method of steepest descents for energy minimization. After energy minimization, the system was simulated for 1 ns at 550 K in the NVT ensemble using the V-rescale thermostat.^71^ The pure PLLA system was then equilibrated at 550 K and 1 bar using the V-rescale thermostat^71^ (with a time constant of 0.1 ps) and the Parrinello–Rahman barostat^72^ for 1 μs. Bonds involving hydrogen were constrained using the P-LINCS algorithm.^73^ After the above procedure, 150, 308, 1000, and 1958 water molecules were randomly added to the polymer matrix to obtain the desired PLLA–water systems of 1, 2, 6, and 12 m-%, respectively. Each of these systems was relaxed and equilibrated following the protocol described above (at 550 K and 1 bar). Three independent replicates (S1, S2, and S3) were prepared separately with this procedure. Each of these replicates with 0, 1, 2, 6, and 12 m-% of water, a total of 15 systems, was equilibrated for 3 μs with a NpT ensemble at a temperature of 300 K and pressure of 1 bar.

### 2.2 Production Systems

To simulate the PLLA–water systems, each of the PLLA–water systems was equilibrated at 300 K after a cycle of relaxation, reheating, relaxation, reheating, and pressurizing at 1 bar. Equilibration for 3 μs was done following the same protocol as described above. The production run was 100 ns for data collection. The annealing simulations were carried out for a fourth set of independent replicates, which had been prepared as above and equilibrated for 1 μs. In all of the above stages, the particle-mesh Ewald^74,75^ (PME) method was used for the long-range Coulomb interactions with a Fourier spacing of 0.16 and cubic splines. The time step was 1 fs, and the van der Waals interactions and the short-range part of the Coulomb interactions were cut off at 1.0 nm. Periodic boundary conditions (PBC) were used in all directions. The long-range dispersion correction was applied for energy and pressure.

To determine the annealing densities of the pure PLLA, and the 1, 6, and 12 m-% PLLA–water systems, the systems equilibrated at 550 K were annealed with a cooling rate of 1 K per 20 ps, i.e., 3 × 10^12^ K/min, from 550 to 100 K within a single cooling simulation; from a temperature exceeding the experimentally known melting temperature to well below *T*_g_. The cooling was carried out through a continuous annealing process.^76,77^ Unlike the stepwise method, annealing does not skip any temperature points.

## 3 Results

### 3.1 Structural Data

Snapshots of the equilibrated PLLA–water systems are presented in Figure 2. They show that the water molecules pair up, or form clusters of three molecules already at 1 m-% of water, and of eight molecules at 2 m-% (Figure 2b,f). The aggregation of water molecules increases with increasing water concentration (Figure 2c,d,g,h). Similar behavior has been reported in the experiments of Cerveny et al., who observed water clusters in the poly­(vinyl methyl ether) (PVME) matrix.^57^ Clustering of water has also been proposed in high *L*-content PLA–water films.^78^ Entrialgo-Castaño et al. found, by MD simulations of PLLA that at 2 m-% more than half, and at 7 m-% all of the water molecules were involved in hydrogen bonding with each other.^79^ Clustering has also been reported in coarse-grained molecular simulations of PDLLA (50% *D*).^47^

**Figure 2:**
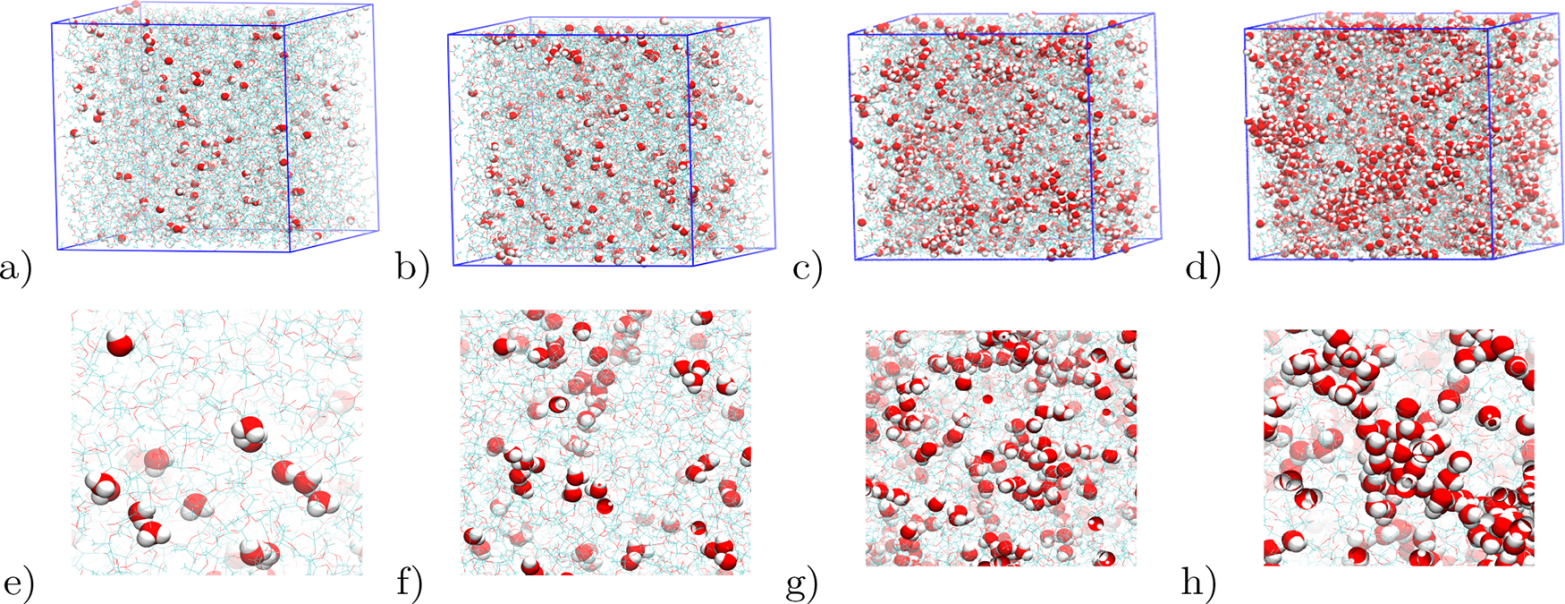
Equilibrated *a*-PLLA chains (25) in a cubic box at 300 K and 1 bar. The PLLA chains are drawn as lines and the water molecules with van der Waals representation (cyan = carbon, red = oxygen, white = hydrogen). (a) With 1 m-% of water (150 H_2_O molecules), (b) 2 m-% of water (308 H_2_O molecules), (c) 6 m-% of water (1000 H_2_O molecules), and (d) 12 m-% of water (1958 H_2_O molecules). (e–h) Close-ups of the same systems, respectively. Note that the 1 m-% is slightly below the experimentally determined equilibrium uptake of water in PDLLA (1.4% at 293 K).^55^

Xiang and Anderson used MD simulations to study water sorption in an amorphous PDLLA.^80^ They compared their results with a water sorption isotherm (which they created from experimental data) at low water sorption, where Henry’s law applies, and concluded that <2% w/w of water sorbed into *a*-PLA, when the water content was (0–100)% relative humidity. However, in experiments both amorphous *a*-PLLA obtained by quenching, and intrinsically semicrystalline *c*-PLLA have been observed to absorb approximately the same amounts of water during the first ca. 20 weeks, i.e., ca. 2% in 2–3 weeks and ca. (5–7)% during 7–12 weeks.^50^ Moreover, the maximum measurable water uptake, which depends on the morphology, is much lower for *c*-PLLA (28% after 70 weeks) than for *a*-PLLA (105% at week 110).^50^ Our water content corresponds to the time period before ca. 20 weeks, after which water absorption, degradation to *L*-lactic acid, and the water-uptake-induced crystallinity content of *a*-PLLA clearly increased^50^ in experiments.

### 3.2 Radial Distribution Functions and Coordination Numbers

The probabilities of finding pairs of atom types *A* and *B* in the adjacent PLLA chains at a distance *r* from each other were analyzed using the radial distribution function (RDF). The RDF between *A* and *B* is defined as

$$g_{AB}(r)=\frac{\left\langle \rho_{B}(r)\right\rangle }{\langle \rho_{B}\rangle_{\mathrm{local}}}=\frac{1}{\langle \rho_{B}\rangle_{\mathrm{local}}}\frac{1}{N_{A}}\sum_{i\in A}^{N_{A}}\sum_{j\in B}^{N_{B}}\frac{\delta (r_{ij}-r)}{4\pi r^{2}}$$ 1

where ⟨ρ_*B*_(*r*)⟩ is the average density of type *B* atoms in the system at a distance *r* around atoms *A*, *N*_*A*_ the number of *A* atoms, ⟨ρ_*B*_⟩_local_ is the atom density of type *B* averaged around atoms *A* with radius *r*_max_ and *r*_*ij*_ is the distance between atoms *A* and *B*, and *δ* is the Dirac delta function.^81^ The number of neighbors can be computed using the cumulative radial distribution function (CRDF),

$$G_{AB}(r)=\int_{0}^{r}dr^{\prime }4\pi r^{\prime 2}g_{AB}(r^{\prime })$$ 2

as the average number of *B* atoms within the radius *r* around atoms *A*. The number of closest neighbors (coordination number), i.e., the first solvation shell can be computed by integrating eq 2 up to the first minimum of *g*(*r*).

#### 3.2.1 Intermolecular Interaction Probabilities between Atom Pairs in the Adjacent Poly­( *l* -lactide) Chains in the Absence and Presence of Water

The contact pairs representing the most prominent interatomic interactions between adjacent PLLA chains were investigated in the absence (0 m-%) and presence of water (1–12 m-%) using RDFs and CRDFs. The nearest-neighbor contacts within the same chain, i.e., within the same CRU, were excluded from the analysis.

The most prominent interaction probability, *g*(*r*), is obtained from the RDF plot at its first maximum, while the number of nearest neighbors, i.e., the coordination number (coord#), is calculated from the CRDF results using eq 2 and integrating to the distance corresponding to the first minimum in the RDF curve. Figures 3 and 4 present the RDF and CRDF plots for six pairs of atoms (see Figure 1 for the atom labels) with the highest *g*(*r*) values, characterized by consistently short interatomic distances at all water contents. The RDF and CRDF plots for the remaining nine atom pairs are provided in Figures S1–S4. The numerical values of *g*(*r*) and coord# are shown in the summary bar charts in Figures 5 and 6, respectively, and are also provided with the standard error of the mean (SEMs) in Tables S1–S3 for all systems. All RDF probabilities converged to 1.0 below 1.0 nm.

**Figure 3:**
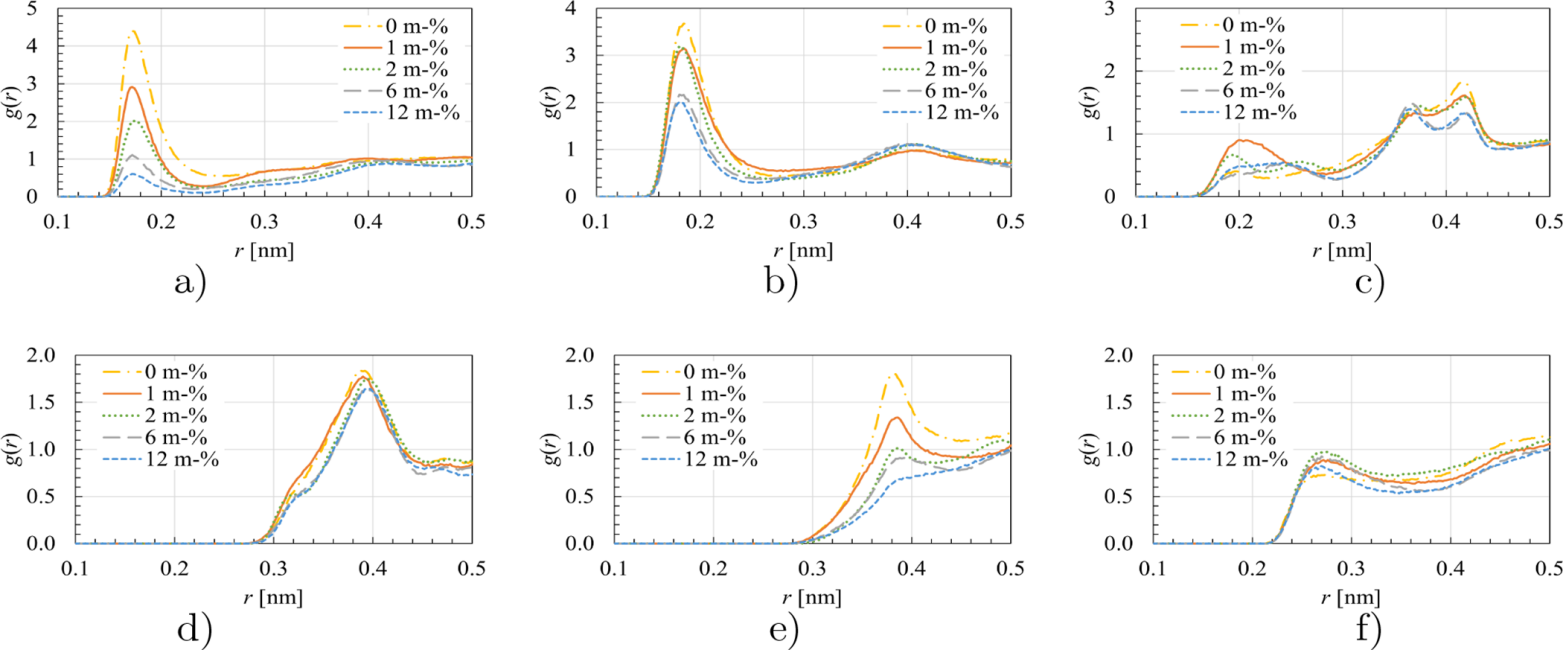
Radial distribution functions for pairs of (a) terminal alcohol hydrogen–carbonyl oxygen (H11–O4), (b) terminal carboxyl hydrogen–carbonyl oxygen (H10–O4), (c) terminal carboxyl hydrogen–ester oxygen (H10–Os) (d) terminal carboxyl oxygen–carbonyl carbon (O1–C2) (e) terminal alcohol oxygen–carbonyl carbon (O10–C2), and (f) terminal carboxyl oxygen– α-hydrogen (O1–H5), in pure PLLA (0 m-%, yellow – ·−) and with 1 (orange solid), 2 (green ··), 6 (gray – −), and 12 (blue short dash line) m-% of water in the PLLA matrix. See Figure 1a) for atom labeling.

**Figure 4:**
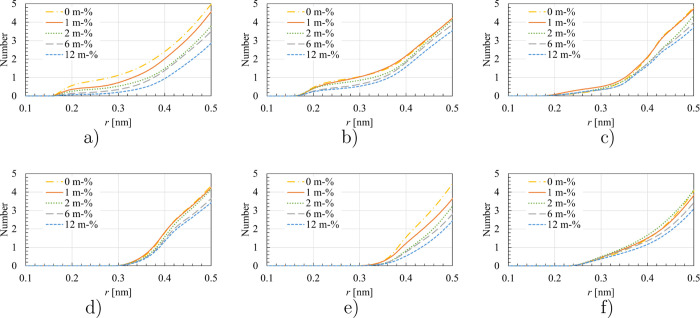
Plots of the cumulative RDFs (CRDFs), coordination number (coord# = Number) as a function of the distance for pairs of (a) terminal alcohol hydrogen–carbonyl oxygen (H11–O4), (b) terminal carboxyl hydrogen–carbonyl oxygen (H10–O4), (c) terminal carboxyl hydrogen–ester oxygen (H10–Os) (d) terminal carboxyl oxygen–carbonyl carbon (O1–C2) (e) terminal alcohol oxygen–carbonyl carbon (O10–C2), and (f) terminal carboxyl oxygen– α-hydrogen (O1–H5), in pure PLLA (0 m-%, yellow – ·−) and with 1 (orange solid), 2 (green ··), 6 (gray – −), and 12 (blue short dash line) m-% of water in the PLLA matrix.

**Figure 5:**
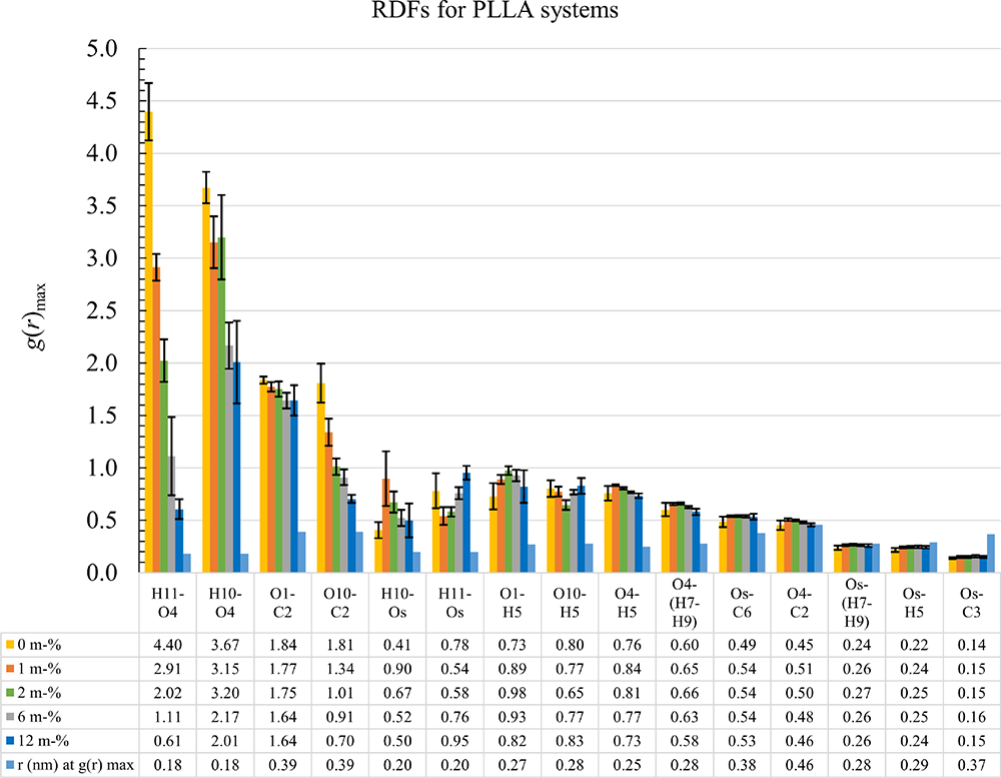
RDF peak maxima, with ± SEM error bars (———), and separation distances *r*(nm) at the peak positions. Data for the pairs H11–Os, O10–H5, O4–H5, O4-(H7–H9), Os–C6, and O4–C2 are provided in Figures S1 and S3, and for all pairs in Tables S1–S3.

**Figure 6:**
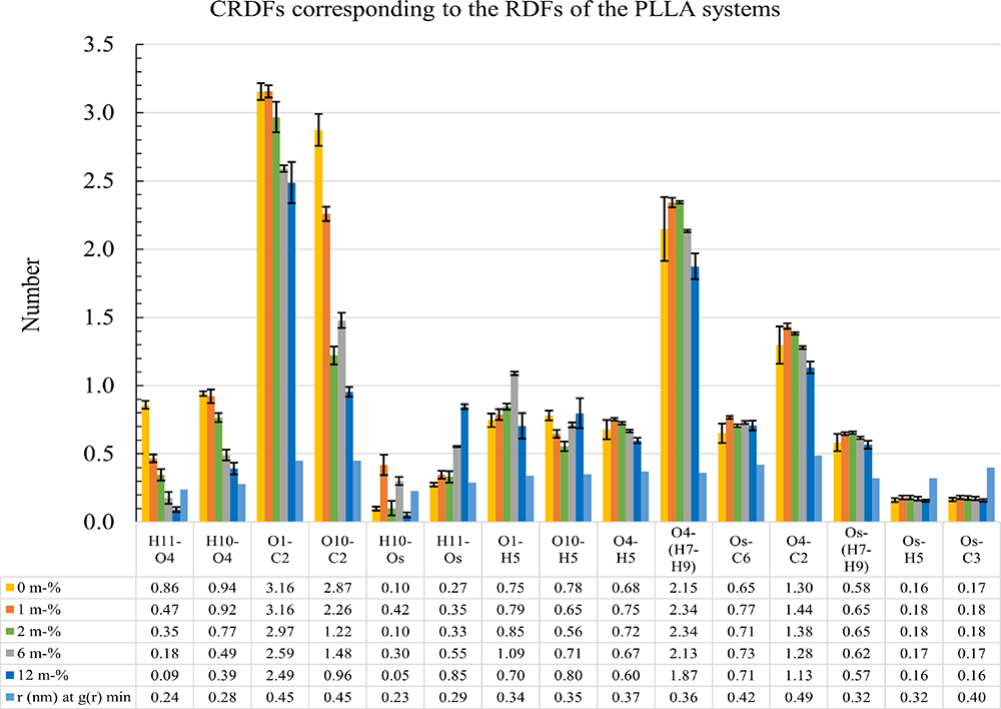
Coordination number at the first RDF minimum, with ± SEM error bars (———), and the position of the first minimum, *r*(nm). Data for the pairs H11–Os, O10–H5, O4–H5, O4-(H7–H9), Os–C6, and O4–C2 are provided in Figures S2 and S4, and for all pairs in Tables S1–S3.

#### 3.2.2 Hydrogens (H11 and H10) of the Terminal Structural Units

We first focus on the hydrogens of the alcohol (OH) (H11) and carboxyl groups (COOH) (H10) at the terminal structural units (see Figure 1 for the labeling of the atoms). Both hydrogens have their strongest RDF peak with a value of *g*(*r*) of approximately 4 at an interatomic distance of around 0.18 nm with carbonyl oxygens (O4) for pure PLLA (0 m-% water) (H11–O4 and H10–O4 pairs in Figures 3a and b, respectively, and Figure 5). Differences appear when 1 and 2 m-% of water is added: for H11–O4 the RDF peak height decreases to ca. 2.9 and 2.0, respectively, but for H10–O4 the peak remains high at 3.2. As more water is added, the peak value of *g*(*r*) drops for H11–O4 to 1.1 at 6 m% and to 0.6 at 12 m%. This can be interpreted as the water strongly interpenetrating between the PLLA chains and reducing the interchain contacts, especially at 12 m-%; the H11–O4 contact is not strong enough to prevent water from entering.

The situation is markedly different for carboxyl hydrogen–carbonyl carbon (H10–O4) at the highest water contents. At 6 m-%, the RDF peak remains relatively high (2.2) and is approximately the same as the RDF peak height of the H11–O4 pairs at 2 m-%. At 12 m-%, the peak decreases, although its value is still around 2.0. In other words, the H10–O4 peak remains much higher and decreases far more gradually than the H11–O4 peak as the water content increases. This indicates that the contacts between the carbonyl oxygens (O4) and the terminal carboxyl hydrogens (H10) are less susceptible to disruption by water. These results agree with organic chemistry knowledge on the reactivity of carbonyl oxygen, which can be activated by an electrophile, such as an acid proton, and with previous reports on the autocatalyzed degradation^1,34,82,83^ reactions of PLLA by acidic COOH hydrogen. The terminal alcohol proton is not as acidic, because the alkoxide group is not stabilized by the neighboring atoms as in the COOH group, and therefore contact with O4 is more easily disturbed by water (Figure 3a and Figure 5).

In the absence of water, the number of nearest neighbors (coord#) is ≈ 1 for both H11–O4 and H10–O4 (Figures 4a and b, and Figure 6). For H11–O4, number decreases to ca. 0.5 at 1 m-% and becomes nearly zero at 12 m-%. These results agree with experimental studies, in which PLLA absorbs water, swells, and begins to degrade, at low water concentrations.^50^ For H10–O4, the number of nearest neighbors is ≈ 1 at 1 m-% and remains around 0.4 at the highest water concentration (Figure 6).

Terminal alcohol hydrogens (H11) and carboxyl hydrogens (H10) form the next closest (0.20 nm) atom pairs within pure PLLA chains with carboxyl oxygens, hereafter referred to as ester oxygens (Os) for clarity. The RDF peak heights are 0.8 (Figure 3c; Table S2) for H11–Os and only 0.4 (Figures S1a and 5; Table S1) for H10–Os, with very low coord#s (0.3 and 0.1, respectively) (Figures 4c, 6 and S2a). When 1-m% of water is added, the H10–Os distance remains at 0.20 nm, and the RDF peak increases to nearly 1. In contrast, the H11–Os distance increases to 0.26 nm, and its RDF peak decreases. The coordination numbers remain low for both contact pairs at 2–6 m-%. However, at 12 m-%, *g*(*r*) increases to ≈ 1 for H11–Os at a distance of 0.30 nm (with coord# ≈ 0.9) (Table S2). These results indicate that H10–Os contact pairs maintain short separations within the PLLA polymer even in the presence of water, that a small amount of water promotes the formation of H10–Os contacts, and that H11–Os contacts become favored at high water concentrations (12 m-%).

#### 3.2.3 Oxygens (O1 and O10) of the Terminal Structural Units

The third and fourth highest RDF peaks (ca. 1.8) are found for pairs of O1–C2 (Figures 3e, 4e, 5 and 6) and O10–C2 atoms (Figures 3d, 4d, 5, and 6) in pure PLLA, with the largest coordination numbers (coord# 3.2 and 2.9, respectively). Although carbonyl carbons (C2) of the PLLA chains, as partially positive, and oxygens of both the terminal carboxyl (O1) and alcohol (O10) groups as partially negative, tend to neighbor each other. The distance (0.39 nm) between these atoms is greater than between the pairs of H11–O4 and H10–O4 discussed above, because the carbon and oxygen atoms have larger van der Waals radii than hydrogen. At 1 m-%, the RDF remains approximately the same as in the pure PLLA for O1–C2, with as high coord# of 3.2. The shapes and heights of the O10–C2 RDF peaks are more strongly affected by water interference: the peaks decrease as the water content increases, although the contact persists up to 6 m-%, whereas the RDF peak of O1–C2 changes only slightly at 6–12 m-% (*g*(*r*) ≈ 1.6; coord# 2.5). These results indicate that the interatomic electrostatic interactions between the polar COOH and C=O groups of the PLLA polyester chains persist across all water concentrations studied in this work, and between the alcohol OH and C=O groups at low water concentrations.

The oxygens (O1) of the terminal carboxyl groups can form contact pairs also with the alpha hydrogens, α-H (H5), of the neighboring PLLA chains at a distance of ca. 0.27 nm (Figures 3f, 4f, and 5). The α-H is acidic, that is, electrophilic by nature because of its proximity to the carbonyl group oxygen that withdraws electrons from the α-carbon and α-H. This contact is the most likely to occur at 2–6 m-% water content.

Additional data for pairs H11–Os, O10–H5, O4–H5, O4-(H7–H9), Os–C6, and O4–C2 are provided in Figures S1, S2, and Table S2, and data for Os-(H7–H9), Os–H5, and Os–C3 in Figures S3, S4, and Table S3. Some of these, such as Os–H5, O4-(H7–H9), and O4–C2 contacts, may play important roles in crystalline structures and in degradation mechanisms,^84–86^ of PLLA, although some of them, including the atom pairs of the end groups H10–O10 and H11–O1, which did not yield a histogram, were found to be less favorable or unfavorable, respectively.

#### 3.2.4 Summary of the RDF and CRDF Results

Overall, the most favorable interchain interactions arise from contacts between the terminal carboxyl OH or alcohol OH groups, and the C=O groups of the neighboring *a*-PLLA backbones with water content of 0–6 m%. In a water-free environment, the terminal carboxyl hydrogens (H10) and the alcohol hydrogens (H11) preferentially interact with the protruding carbonyl oxygen atoms (O4) of the PLLA chains. With water, contacts are more likely between H10 and O4 than between H11 and O4 at 1–12 m-%. However, the coordination number decreases below 1 in both systems. The results correlate with experiments on the autocatalyzed degradation of PLLA by acidic COOH hydrogen at low water concentrations.^50^

In pure PLLA and with a low water content (1–6 m-%), the terminal carboxyl oxygens (O1) and the alcohol oxygens (O10) are the third and fourth most probable atoms, respectively, to form contact pairs, namely with carbonyl carbon atoms (C2) of adjacent polymer chains. The O1–C2 contacts are more likely, and they also remain more frequent than contacts between alcohol hydrogens (H11) and carbonyl oxygens (O4) at higher water concentrations (6–12 m-%), although the atoms in the O1–C2 pairs are farther apart than in the H11–O4 pairs. For the carboxyl oxygen - carbonyl carbon contact (O1–C2), the coordination number is the highest (2.5–3.2) across all systems studied, despite the presence of water. The results indicate that the interatomic electrostatic interactions between the polar OH and C=O groups of the PLLA polyester chain persist at low water content, and better with the COOH group.

In the case of contacts between terminal H10 and H11 hydrogens with backbone ester oxygens, Os, water decreases the probability of H11–Os atom contacts compared to contacts within the water-free PLLA but increases the likelihood and coordination number of H10–Os atom contacts at low water concentrations. At higher water concentrations, H11–Os contacts and coordination become more probable. The results correlate with experimental studies by Li et al., in which water absorbs into PLLA and starts to degrade, faster in the bulk than on the surface.^50^ This can be interpreted that during swelling of the PLLA matrix, water separates the chains from each other, which promotes the beginning of degradation of the material. Degradation is expected to enable additional atomic contacts at elevated temperatures, e.g., by increasing the number of OH groups, which are produced in the hydrolysis of PLA.^87^

Close contacts between terminal carboxyl oxygen (O1) and acidic α-hydrogens (H5) are also unhindered and persist at all concentrations studied. In addition, O10–H5 contact pairs can form in the presence of water, but with a lower probability than the O1–H5 pairs. Water slightly increases all RDF peak intensities of the O1–H5 pairs compared to pure PLLA.

Carbonyl oxygen, O4, can also be in close contact with α-hydrogens (H5) or methyl hydrogens (H7–H9), i.e., β- hydrogens, of adjacent chains. The addition of water increases the coordination number from that of pure PLLA for both contacts (Figure 6) at 1 and 2 m-%. Although *g*(r) is only ca. 0.6–0.8, the increase in coord# with added water could be correlated with the experimental observation that water increased the crystalline content of *a*-PLLA.^50^ On the other hand, the transfer of β–C-H hydrogen to carbonyl oxygen (O4) has previously been proposed as a step in the degradation mechanism of PLA, which produces shorter carboxylic acid fragments.^86,88^ Moreover, a degradation mechanism through trans-esterification and cyclization reactions has been proposed^86^ to occur between ester oxygens (Os) and α-carbons (C3), but this contact is predicted to be highly unlikely in our study.

## 4 Thermodynamic and Mechanical Properties

Next, we analyze the mechanical and thermodynamic properties of the pure *a*-PLLA and *a*-PLLA–water systems (Tables 1 and 2). Two simulations with 100% water are provided for comparison, one with 13 335 molecules and another with 1958 molecules, the same amount as in the 12 m-% system.

**Table 1:** Average Values of Simulation Box Volumes (*V*), Thermal-Expansion Coefficient (α_p_), Isothermal Compressibility (β_T_), and Isothermal Bulk Modulus (*K*_T_) for Pure PLLA, PLLA–water systems with 1, 2, 6, and 12 m-% of water, and pure water at 300 K and 1 bar^a^

H_2_O [m-%]	*V* [nm^3^]	*V* × 10^–5^ [m^3^ mol^–1^]	α_p_ × 10^–4^ [1/K]	β_T_ × 10^–10^ [Pa^–1^]	*K*_T_ × 10^9^ [Pa]
0	374.6 ± 5.4	6.02 ± 0.09	3.96 ± 0.15	4.43 ± 0.14	2.26^d^
1	373.4 ± 0.1	6.00 ± 0.01	3.96 ± 0.06	4.29 ± 0.05	2.33
2	376.8 ± 0.2	6.05 ± 0.01	3.93 ± 0.06	4.21 ± 0.13	2.38
6	392.0 ± 0.9	6.30 ± 0.02	4.41 ± 0.15	4.07 ± 0.12	2.46
12	418.1 ± 0.5	6.72 ± 0.01	5.48 ± 0.29	4.19 ± 0.07	2.39
100^b^	59.5 ± 0.4	1.83	9.21	6.08	1.64
100^c^	405 ± 1	1.83	9.34	6.09	1.64
ref. H_2_O 298 K, 1 bar	-	-	2.56 ^94^	4.52 ^94^	-
ref. H_2_O 303 K, 1 bar	-	-	3.02 ^94^	4.48 ^94^	-
ref. PLA ≈ 310 K	-	-	2.4–4.6 ^93^	-	-
ref. PLA melt, 1 bar	-	-	-	9.0–11.8 ^93^	-
PLA solid, 1 bar	-	-	-	2.7–5.9 ^93^	-

a Calculated values from three independent replicate system groups (S1, S2, and S3) were averaged and the fluctuation ranges were calculated as SEM. System specific values are available in Table S4. Properties for pure water and PLA are given as references.

b Simulated water system with 1958 water molecules, the same amount as in the 12m-% PLLA-water system.

c Simulated system with 13 335 water molecules for a total volume comparable to the PLLA systems.

d *K*_T_ = 1.52 GPa, estimated from experimental values from ref 2. using eq 6 for PLLDA (*L*:*D* 96:4).

**Table 2:** Thermodynamic Properties for Pure PLLA, PLLA–Water Systems with 1, 2, 6, and 12 m-% of Water, and Pure Water at 300 K and 1 bar: Enthalpy of Vaporization, *H*, Molar Isobaric (J mol^–1^K^–1^) and Specific Heat Capacity (J g^–1^K^–1^), *C*_p_, and the Difference *C*_p_ – *C*_V_ between *C*_p_ and the Heat Capacity at Constant Volume, *C*_V_ ^f^

H_2_O [m-%]	*H* [kJ mol^–1^]	*H* [kJ kg^–1^]	*C*_p_ [J mol^–1^ K^–1^]	*C*_p_ [J g^–1^ K^–1^]	*C*_p_ – *C*_V_ [J mol^–1^ K^–1^]	*C*_p_ – *C*_V_ × 10^–3^ [J g^–1^ K^–1^]
0	123.5 ± 0.3	1710 ± 5	203 ± 1	2.81 ± 0.02	6.39 ± 0.54	8.85 ± 0.74
ref.	-	-	5–250 ^21^	-	-	-
-	-	-	95.30^a^ ^99^	-	-	-
1	116.9 ± 0.1	1685 ± 1	198 ± 1	2.83 ± 0.01	6.32 ± 0.13	9.01 ± 0.19
2	110.7 ± 0.3	1660 ± 5	192 ± 1	2.82 ± 0.02	6.17 ± 0.24	9.06 ± 0.36
6	89.2 ± 0.1	1566 ± 1	179 ± 2	2.94 ± 0.03	7.13 ± 0.28	11.72 ± 0.46
12	68.3 ± 0.1	1440 ± 1	167 ± 1	3.11 ± 0.01	9.50 ± 0.84	17.72 ± 1.58
100^b^	32.5 ± 0.2	1804	79.8	4.43	7.66	42.5
100^c^	32.5 ± 0.2	1804	79.4	4.41	7.86	43.6
ref.^d^	42.87^d^	2382^d^	75.3^e^	4.18^e^	0.909^e^	5.04^e^
TIP3P	-	-	75 ^100^	4.16 ^100^	-	-

a PLLA (1.5% D), *M*_w_ 180,000–220,000g/mol, 300 K.^99^

b Simulated water system with 1958 water molecules, the same amount as in the 12 m-% PLLA-water system.

c Simulated system with 13,335 water molecules for a total volume comparable to the PLLA systems.

d Experimental enthalpy of vaporization of water at 300 K and 0.124 bar.^107^

e Experimental values for water at 300 K and 1 bar: *C*_p_: 75.315 J/(molK); *C*_V_: 74.406 J/(molK): *C*_p_ – *C*_V_ = 0.909 J/(molK);^101^ *C*_p_: 4.1806 J/(gK), *C*_V_: 4.1302 J/(gK): *C*_p_ – *C*_V_ = 0.0504 J/(gK).^107^

f Calculated values from three independent replicate system groups (S1, S2, and S3) were averaged and the fluctuation ranges were calculated as SEM. System specific values are available in SI Table S5. Properties of pure water are given for reference.

### 4.1 Volumetric Dilation

When water is added to the *a*-PLLA matrix, the box size first decreases only slightly at 1 m-% and then increases, Table 1. The corresponding changes in densities are discussed in detail in Section ’Densities’ (Table 3). The addition of 1 m-% water results in a contraction of ca. 0.3% and 2 m-% results in an expansion of 0.6% in volume, respectively. After that, the system undergoes a small but systematic volumetric dilation (volumetric swelling) with a further addition of water. At 6 m-% and 12 m-% the volume has expanded 5% and 12% from the original pure *a*-PLLA volume (0 m-% water), respectively (Table 1).

**Table 3:** Average Densities (ρ) of the Pure PLLA and PLLA–Water Systems with Different Mass Percentages of Water, H_2_O [m-%], Simulated at 300 K and 1 bar and Annealing Scans Simulated from 550 to 50 K at 1 bar^a^

H_2_O [m-%]	simulation ρ [kg m^–3^]	annealing ρ [kg m^–3^]	Exp. ρ [kg m^–3^]
0	1200 ± 17	1198 ± 3	MAF 1240,^102^ RAF *α′* 1170,^102^ and RAF α 1100 ^102^ (PLLA at 294 K̇); 1152 ^65^ (PLA at 413 K); 1248 ^52^ (*a*-PDLLA); α 1245 ^102^ and *α′* 1265 ^102^ (*c*-PLLA at 294 K̇); single cryst. 1290 ^52^ (PDLLA)
1	1216 ± 1	1201 ± 2	-
2	1217 ± 1	-	-
6	1223 ± 3	1198 ± 3	-
12	1215 ± 1	1194 ± 2	-
100^b^	984 ± 6	-	996.56 ^107^
100^c^	984 ± 3	-	-
TIP3P^d^	982 ^63^	-	-

a The range of simulation density was calculated as SEM from the independent replicate systems. The values for the annealing densities were calculated as an average of 30 data points above and 30 points below 300 K and error bars as RMSD. Experimental values from literature are provided in the last column. MAF: Mobile amorphous fraction; RAF: rigid amorphous fraction.

b Simulated water system with 1958 water molecules, the same amount as in the 12 m-% PLLA-water system.

c Simulated system with 13,335 water molecules for a total volume comparable to the PLLA systems.

d 298 K.

The uptake of water and/or the volumetric swelling have been experimentally studied for PLA and its copolymers and PDLLA: Gilding and Reed reported very low water uptake for crystalline PLA and its copolymer in a 0.2 M pH 7 buffer during 3–4 days.^89^ Van de Witte et al. observed swelling of ca. 1.4% in water^90^ for amorphous PDLLA films; for crystalline PLLA swelling was lower and could not be determined. Li et al. reported that the absorption of water was slow for amorphous poly­( *l* -lactic acid) during the first 12–18 weeks in pH 7.4 buffer;^50^ material with low molecular weight and crystallinity emerged almost within the same time frame, after 12 weeks, and they concluded that chain relaxation during water uptake and degradation was the cause.^50^ The crystalline parts that formed were highly resistant to degradation.^50^ After 18 weeks, water absorption increased over 10% and *L*-lactic acid monomers clearly began to be released. Semicrystalline PLLA absorbed less water.^50^ The water penetration, swelling, and resistance to swelling due to water at low concentrations have also been studied with a microbalance in PDLLA–water systems.^55^

Entrialgo-Castaño et al.^79^ simulated PLLA only at two water contents (2 and 7 wt %) and calculated volumetric swellings compared to dry PLLA. However, their model at 310 K, using the polymer consistent force field (PCFF), was smaller than ours, consisting of 19 chains of 50 repeating units, and they did not study contacts between the polymer chains. In addition, the final dimensions reported for their models^79^ first decrease at 2 wt % and then increase, as the density first increases and then decreases already at 7 wt %, which could be due to initial contraction and subsequent dilation, when more water is added to the system, but they did not, however, comment on this. However, the values calculated for volumetric contraction and swelling, from their volumes, are very small. In shape-memory studies of *a*-PLLA using MD with the COMPASS force field, a single 720 CRU-long dry PLLA model and the models with 1 and 2 wt % of water had similar shape memory properties.^91^ The density first increased at 1 wt % and then decreased already at 2 wt % at 500 K.^91^ At 300 K, MD-simulated volumetric dilation of ca. 2% was obtained with 2 wt %.^91^ Variations in the structures produced by the different models, only two concentrations of water, different force fields, and the distribution of the free volume elements have led to different results. To our knowledge, the current study is the first to invstigate the swelling behavior of PLLA in detail.

### 4.2 Thermal-Expansion Coefficient

The isobaric, volumetric coefficient of thermal expansion is defined as

$$\alpha_{p}=\frac{1}{V}{\left(\frac{dV}{dT}\right)}_{p}$$ 3

where *V* is the volume *T* is the temperature, and *p* is the pressure. In practice, α is calculated as

$$\alpha_{p}=\frac{\left\langle VH\rangle -\langle V\rangle \langle H\right\rangle }{\langle V\rangle T^{2}k_{B}}$$ 4

where the angular brackets denote an average. The enthalpy, *H*, is calculated by *H* = *E*_tot_ + *pV*, where *E*_tot_ is the total energy of the system. The simulations predict the α_p_ values excellently (Table 1) compared to typical experimental values for solids and liquids^92^ and especially for solid PLA.^93^ Pure simulated PLLA and PLLA with 1 m-% of water have an α_p_ of 3.96 × 10^–4^ K^–1^, which decreases very slightly to 3.93 × 10^–4^ K^–1^ with 2 m-% of water likely due to contraction of the system when water comes into contact with the PLLA chains. Upon the addition of more water, α_p_ increases to 4.41 × 10^–4^ K^–1^ at 6 m-%, reaching 5.48 × 10^–4^ K^–1^ at 12 m-%, as more water penetrates between the PLLA chains and the system expands, that is, the polymer matrix swells.

### 4.3 Isothermal Compressibility and Isothermal Bulk Modulus

The isothermal compressibility defined as β_T_ = −*V* ^–1^(*∂V*/*∂p*)_T_ was computed as^95^

$$\beta_{T}=\frac{\left\langle V^{2}\right\rangle -{\left\langle V\right\rangle }^{2}}{k_{B}\left\langle T\right\rangle \left\langle V\right\rangle }$$ 5

For pure PLLA at 300 K, the simulations yielded β_T_ = 4.43 × 10^–10^ Pa^–1^, which is within the range of experimental values of (2.7–5.9) × 10^–10^ Pa^–1^ reported for solid PLA (Table 1).^93^ Addition of water first decreased the compressibility with β_T_ reaching its lowest value of 4.07 × 10^–10^ Pa^–1^ at 6 m-%. Further addition of water increased β_T_ to 4.19 × 10^–10^ Pa^–1^ at 12 m-%, i.e., to about the same level as in the 2 m-% system.

The inverse of the isothermal compressibility yields the isothermal bulk modulus, *K*_T_ = β_T_^–1^. For pure PLLA (Table 1), the simulations yielded *K*_T_ = 2.26 GPa.

Using *K*_T_ together with data for high *L*-content PLLDA (*L*:*D* 96:4) with *M*_w_ = 66 kg/mol in Farah et al.,^2^ the experimental isothermal bulk modulus can be determined using the Lamé relation

$$\nu \approx \frac{1}{2}-\frac{E}{6K_{T}}$$ 6

where ν is the Poisson ratio, *E* is Young’s modulus, and *K*_T_ is the bulk modulus at constant temperature. Solving for *K*_T_, and substituting for values ν = 0.36, and *E* = 1.28 GPa (Table 1 in ref 2) gives *K*_T_ = 1.52 GPa. Very similar experimental Young’s moduli (i.e., tensile moduli) of 1.3–1.5 GPa have also been reported for P­(*L*,*D*)­LA (96% *L*) and P­(*L*,*DL*)­LA (70% *L*).^19,20^ For dry *c*-PLLA, higher *E* values of 2.7–4.14 GPa^96^ (Table 2 in ref 2) have been measured. Hygrothermal aging at 293 K has increased the *E* value of *c*-PLLA from 3.66 to 3.68 GPa (as reported in Table 13 of ref 2), which reflects an increase also in the isothermal bulk modulus. Thus, our simulations predict the bulk modulus for pure *a*-PLLA in very good agreement with the experiments. A direct comparison with an experimental or simulated bulk modulus of *a*-PLLA at 300 K was not possible because, to our knowledge, no values have been reported in the previous literature.

When water was added to the PLLA matrix model, *K*_T_ increased to 2.33 GPa at 1 m-%, and reached the maximum of 2.46 GPa at 6 m-%. At 12 m-% *K*_T_ decreased to 2.39 GPa, which is about the same as at 2 m-% (Table 1). The isothermal bulk modulus *K*_T_ of pure water calculated from the experimental isothermal compressibility β_T_ at 298 K (Table 1, ref 94) is ca. 2.21 GPa, which is closer to the simulated *K*_T_ values of the PLLA and PLLA–water mixtures than to that of the 100% water system (Table 1).

When comparing results between experiments and simulations, it is important to note that the commonly used TIP3P model of water, which is also used here, has *K*_T_ ≈ 1.74 GPa at 298 K^97^ and should be used as a reference for the simulation. The TIP3P somewhat underestimates *K*_T_. The use of a better model for water such as TIP4*P*/2005 ^98^ would be desirable (*K*_T_ ≈ 2.15 GPa at 298 K), but that would require compatibility with the polymer force field.

Plasticizers and swelling tend to decrease mechanical strength and moduli.^2,50^ This is reflected in the reduction of the moduli of the PLLA–water system at the highest water contents (Table 1). In the experiments of Tsuji and Sumida,^49^ as-cast PLLA films were exposed to organic, nonaqueous solvents. That led the Young’s modulus to decrease from the original 0.796 GPa (81.2 kg/mm^2^) to 0.539–0.755 GPa (55–77 kg/mm^2^) due to swelling. As indicated above by swelling, and demonstrated by the RDF results, the atoms of the PLLA chains slide further apart when water is added; at 12 m-% compression is technically slightly more feasible, and thus the bulk modulus decreases from the value at 6 m-%.

### 4.4 Heat Capacity

The isobaric heat capacity is defined as *C*_p_ = (*∂H*/*∂T*)_p_, where *H* is enthalpy. *C*_p_ is obtained from the fluctuations of enthalpy as

$$C_{p}=\frac{\left\langle H^{2}\right\rangle -{\left\langle H\right\rangle }^{2}}{N_{A}k_{B}T^{2}}$$ 7

where *N*_A_ is the Avogadro’s constant and *k*_B_ the Boltzmann constant. The isochoric heat capacity is defined as *C*_V_ = (*∂U*/*∂T*)_V_, where *U* is the internal energy. In NpT simulations, *C*_V_ is obtained from the heat capacity difference as^92,99^

$$\Delta C_{p}=C_{p}-C_{V}=N_{A}\langle V\rangle \langle T\rangle \frac{\alpha_{p}^{2}}{\beta_{T}}$$ 8

The simulated enthalpy of the PLLA system decreases as the water content increases, Table 2. The reference molar *C*_p_ of pure water from our simulations, 79.4 J/(mol·K) (Table 2), is comparable to the reported experimental value of 75.39 ± 0.05 J/(mol·K) at 300 K. Both the simulated molar and specific *C*_p_ of pure *a*-PLLA were higher (Table 2) than the experimental values found for PLDLA: 95.30 J/(mol·K) (with 1.5% *D*) at 300 K^99^ and 1.59 J/(g·K) (with 96:4 *L*:*D*) at 328 K.^2^ However, a wide range of molar *C*_p_ values have been reported for both semicrystalline (190–470 J/(mol·K)) and amorphous PLLA (5–250 J/(mol·K)),^21^ and our simulated values are within this range. The simulated molar *C*_p_ decreases and the specific *C*_p_ increases as the water concentration of the PLLA–water systems increases, Table 2.

PLLA is in its glassy, solid state at 300 K. Heat, which weakens the interatomic interaction forces between the chains, is required to make the amorphous, glassy material rubbery and fluid. Water breaks the interpolymer interactions, facilitating fluidization of the glassy regions and therefore the enthalpy of vaporization decreases. Both the molar and specific heat capacities at (1–12) m-% are gradually approaching the values of pure water.^107^

### 4.5 Densities

The densities (ρ) of the *a*-PLLA and *a*-PLLA–water systems are presented in Table 3. The density of pure *a*-PLLA is below the values of 1.24–1.30 g cm^–3^ reported for semicrystalline PLLA^2^ (Table 3), as expected. The density obtained by the annealing scan at 300 K is between that determined experimentally for the mobile amorphous fraction (MAF) and the rigid amorphous fraction (RAF)^103^ but closer to the lower density found in the RAF structures (Table 3). RAF is typically defined as the region with nanometric dimensions and reduced mobility between the amorphous MAF and the crystalline phase, and is connected to the conformationally disordered modification, i.e., *α′* crystals.^102^ The RDF and density results suggest that there are regions with reduced mobility and stronger contacts between the *a*-PLLA chains. Also, the density of our annealing results at 336 K, which is the experimental *T*_g_ of *a*-PLLA,^78^ is 1187 kg m^–3^, very close to the experimental RAF result.

As Table 3 shows, increasing the water concentration increases the density. This occurs due to the small size of the water molecules, which pack densely among the polymer chains. In addition, in the 1 m-% PLLA–water system, the volume slightly contracts (Table 1), further increasing the density. However, at 12 m-%, the density decreases from that of 6 m-% close to that of 1 m-% as swelling takes place and the total volume increases as a result. In addition, on the basis of the analysis of the RDFs in Section ’Radial Distribution Functions and Coordination Numbers’, the water molecules push the PLLA chains apart. Swelling due to water absorption in PLLA has been, in fact, observed both in aging experiments in aqueous media^50^ and MD simulations at two water concentrations (2 and 7 wt %);^79^ in experiments, hydrolytic degradation (at 310 K) has been reported to start when water concentration increases to ca. 6 – 12 m-%.^50^ MD simulations, however, do not include the possibility to model decomposition or degradation; moreover, the effect of water on the interatomic interactions between the *a*-PLLA chains was not studied by MD in ref 79.

Entrialgo-Castaño et al.^79^ performed MD simulations of smaller *a*-PLLA models consisting of 19 chains, each 50 CRUs long using COMPASS and PCFF force fields at 310 K. They obtained dry densities of about 1220 and 1234 kg/m^3^, respectively. Although our results for the dry system are closer to the experimental RAF results, those of Entrialgo-Castaño et al.^79^ with a 1.25 g/cm^3^ initial density are closer to the MAF results (see Table 3 for experimental values) at 300 K. They did not report the densities for their 2 and 7% water containing systems. Wang et al.^104^ simulated PLA with water (1.6–50 wt %) in 310 K with a polymer model consisting of three 500 CRUs long chains using the PLAFF3 ^105^ force field. However, they did not report the density of the pure PLA or the ratio of *D*- and *L*-isomers or tacticity in their PLA model or the changes in the intermolecular chain interactions. The density at the lowest water concentration of 1.6 wt % was 1.18 g/cm^3^. They reported that the density increased to a maximum 1.19 g/cm^3^ at 6.26 wt %, which is in close agreement with our results. The density decrease was negligible up to 16.7 wt % of water (1.18 g/cm^3^). There was a systematic decrease in density as the amount of water increased to 50 wt %, where it reached 1.09 g/cm^3^. Our simulations are in agreement with these previous findings.

## 5 Conclusions

In this study, we used all-atom MD simulations to explore changes in structural polymer - polymer interactions, as well as thermodynamic and mechanical properties of the *a*-PLLA polymer without and in the presence of varying water concentrations. We note that the convergence of macroscopic observables, volume, density, thermal expansion coefficient, compressibility, and bulk modulus, across replicas combined with the small SEMs for the local structural quantities, provides strong evidence that the reported trends are robust and reproducible within the accessible region of the glassy energy landscape. In particular, the principal trends we discuss, such as the pronounced decrease of the H11-O4 RDF peak from ≈4.4 to ≈0.6 across the water concentration range, or the persistence of the H10–O4 contacts at ≈2.0 even at 12 m-%, involve changes that substantially exceed the inter-replica variability documented in the SI. Our findings reveal several key insights:

### 5.1 Interchain Contacts

The general trend is that as the water concentration increases, the number of contacts between the PLLA chains decreases. Small amounts of water appear, however, to help maintain, or even slightly increase, some contacts between the oxygen and hydrogen atoms of the adjacent *a*-PLLA chains. This is especially the case for the contacts of the chain-end carboxyl OH hydrogens with the ester oxygens, and the carboxyl OH oxygens with the α–hydrogens. In addition, the carbonyl oxygens remain in contact with the carboxyl OH hydrogens, α-hydrogens, and the β-hydrogens of the methyl groups at the lowest water concentrations. The most probable contacts are between the carbonyl oxygens and the chain-end OH hydrogens, but carbonyl carbons are also found to favor several contacts with the chain-end OH oxygens. Electrostatic contacts between these atoms are consistent with forces that would stabilize and maintain local crystalline domains in polymers despite the low amounts of water.

The above results are in agreement with previous reports. In particular, the established autocatalyzed hydrolysis mechanism of esters and polyesters^18^ starts with the transfer of a COOH hydrogen to a carbonyl oxygen. According to our simulations, at low water concentrations this contact is slightly more probable than a contact with an alcohol OH hydrogen, which is also possible. Furthermore, in experiments small amounts of water and degradation have been found to induce crystallinity in *a*-PLLA.^50^ Our simulations show that the number of contacts between COOH hydrogens and ester oxygens, and also between C=O groups, increases at the lowest water concentrations. Moreover, β–C-H hydrogen transfer to carbonyl oxygen has been proposed as one step of the degradation mechanism of PLA.^86,88^ Our simulations find the β–C-H contact almost as probable as the α–C-H hydrogen contact with carbonyl oxygen, and a small amount of water appears to help this contact. However, more neighbors can be found around carbonyl carbons in the case of β-hydrogens. The results highlight the role of water in modulating interchain interactions.

### 5.2 Volume and Thermal-Expansion Coefficient

The reduction in probability and the number of contacts between adjacent chains are correlated with the swelling of the *a*-PLLA-water system at the highest water concentrations of 6 – 12 m-%. At 12 m-% swelling reaches 12% compared to the water-free system. The swelling is in agreement with the experimental data and equilibrium thermodynamics: the amount of absorbed water increases in PDLLA (50:50) as the relative humidity increases.^78,80^ However, at the lowest concentration of 1 m-%, the PLLA matrix model contracts 0.3%, which can be related to increased polymer - polymer interactions and correlated with the experimental findings of increased crystalline domains at low water concentrations.

The simulated contraction and swelling are reflected in the coefficient of thermal expansion. The α_p_ decreases ca. 1% by 2 m-%, as the contacts between the PLLA chains increase slightly. At 6 m-%, α_p_ had increased by 11% as more water molecules permeate between the PLLA chains, which slide farther apart. At 12 m-%, α_p_ increased buy 39%.

### 5.3 Compressibility and Bulk Modulus

Water decreases the compressibility of the PLLA matrix. The simulated bulk modulus, *K*_T_, of pure *a*-PLLA is slightly higher than the value we estimate from the experimental Young’s modulus and Poisson’s ratio of a high *L*-content PLLDA. The value of *K*_T_ increases with water to a maximum of 2.46 GPa at 6 m-%, where the compressibility has its lowest value. But *K*_T_ then decreases to 2.39 GPa at 12 m-%. The experimental bulk modulus of water, 2.2 GPa, is closer to the simulated *K*_T_ values of the PLLA–water mixtures than that of the water-free PLLA. The experimental and simulated isothermal compressibility are higher for pure water than for any of the simulated polymer systems. Plasticizers and swelling are inclined to decrease the moduli and weaken the mechanical properties of polymers as the flexibility of the chains increases at high water content. Hydrolytic degradation (at 310 K) has been experimentally observed at ca. 6 – 12 m-%. It is important to note, however, that while correlations can be drawn between experimentally observed hydrolytic degradation, MD simulations cannot be used to directly model it.

### 5.4 Enthalpy and Heat Capacity

Enthalpy decreases when the water content increases. Water breaks the interpolymer interactions, facilitating fluidization of the glassy regions, and therefore the heat required to break the intermolecular contacts decreases. The simulated heat capacity, *C*_p_, of pure *a*-PLLA is high compared to the experimental value, although also higher values have been reported. The molar and specific heat capacities of water are excellently predicted. The specific heat capacity increases when water is added to the *a*-PLLA–water model matrix and approaches that of pure water.

### 5.5 Density

The density of pure *a*-PLLA obtained at 300 K through an annealing scan is between that determined experimentally for MAF and RAF,^102^ but closer to the lower density of the RAF structure. This result suggests that the PLLA chains are less mobile, in close contact, and organized in some regions, as also predicted by the RDF results. The density (1187 kg m^–3^) simulated by annealing in the experimental *T*_g_ of 336 K is also very close to the experimental RAF density^102^ and previous simulations with long polymer chains.^106^ Water increases the density of the PLLA matrix, as water molecules pack densely among the polymer chains per unit volume. In the 1 m-% PLLA–water system, the volume contracts, further increasing the density. At 6 m-% the density reaches its maximum level. At 12 m-% the density decreases from that of 6 m-% as swelling takes place and the total volume of the model increases, because the water molecules push the PLLA chains apart.

## Supplementary Material



## Data Availability

Data and parameters are available at https://zenodo.org/communities/softsimu.

## Notes

Figure S1: Radial distribution function plots for H11–Os, O10–H5, O4–H5, O4-(H7–H9), Os–C6, and O4–C2; Figure S2: cumulative radial distribution function plots for H11–Os, O10–H5, O4–H5, O4-(H7–H9), Os–C6, and O4–C2; Figures S3 and S4: radial and cumulative radial distribution function plots for Os-(H7–H9), Os–H5, and Os–C3; Figures S5 and S6: volumes during the equilibrations of the replicates for pure PLLA and the 2-m% system; Figure S7: RDFs of the three independent replicate systems for the H11–O4 atom pair; Table S1: numerical values of the RDF and CRDF calculations corresponding to Figures 3 and 4; Table S2: numerical values of the RDF and CRDF calculations corresponding to Figures S1 and S2; Table S3: numerical values of the RDF and CRDF calculations corresponding to Figures S3 and S4; Table S4: average values of the volumes (*V*) of the simulation box, the thermal-expansion coefficient (α_p_), the isothermal compressibility (β_*T*_), and the isothermal bulk modulus (*K*_*T*_); and Table S5: thermodynamic properties for PLLA systems with 0, 1, 2, 6, and 12 m-% of water (PDF)
