# Supplementary material for: Molecular Dynamics Simulations of the Structural and Thermodynamic Properties of Poly( l ‑lactic acid) in the Presence of Water
Source: Macromolecules. 2026 Jun 1;59(12):7199–212. doi: 10.1021/acs.macromol.6c00782 (PMC13296490; doi:10.1021/acs.macromol.6c00782)
Supplement: Supplementary file 1 [file ma6c00782_si_001.pdf]

Molecular Dynamics Simulations of the Structural and Thermodynamic Properties of Poly(L-lactic acid) in the Presence of Water. [†]

Kalle Tuomi,[‡,¶] Terttu I. Hukka,[*,‡] Jurkka Kuusipalo,[¶] and Mikko Karttunen[*,§,‖,⊥,#,@]

‡*Faculty of Engineering and Natural Sciences, Materials Science and Environmental Engineering, Chemistry & Advanced Materials, P.O. Box 541, FI-33014 Tampere University, Finland*

¶*Faculty of Engineering and Natural Sciences, Materials Science and Environmental Engineering, Paper Converting and Packaging Technology, P.O. Box 589, FI-33014 Tampere University, Finland*

§*European Laboratory for Learning and Intelligent Systems (ELLIS) Institute Finland, Maarintie 8, 02150 Espoo, Finland*

‖*Department of Technical Physics, University of Eastern Finland, P.O. Box 1627, FI-70211 Kuopio, Finland*

⊥*Department of Physics and Astronomy, The University of Western Ontario, 1151 Richmond Street, London, Ontario N6A 3K7, Canada*

#*Department of Chemistry, The University of Western Ontario, 1151 Richmond Street, London, Ontario, N6A 5B7, Canada*

@*Department of Physics, P.O. Box 600, FI-33014 Tampere University, Finland*

E-mail: terttu.hukka@tuni.fi; mikko.karttunen1@uef.fi

[†]Electronic supplementary information (ESI) available. See https://doi.org/10.1021/acs.macromol.6c00782

Contents

Figure S1: Radial Distribution Function Plots for H11-Os, O10-H5, O4-H5, O4-(H7-H9), Os-C6, and O4-C2 . . . . . S3

Figure S2: Cumulative Radial Distribution Function Plots for H11-Os, O10-H5, O4-H5, O4-(H7-H9), Os-C6, and O4-C2 . . . . . S4

Figures S3 and S4: Radial and Cumulative Radial Distribution Function Plots for Os-(H7-H9), Os-H5, and Os-C3 . . . . . S4

Figure S5 and S6: Volumes During the Equilibrations of the Replicates for Pure PLLA and the 2-m% System S6

Figure S7: RDFs of the Three Independent Replicate Systems for the H11-O4 Atom Pair S6

Table S1: Numerical Values of the RDF and CRDF Calculations Corresponding to Figures 3 and 4 . . . . . S8

Table S2: Numerical Values of the RDF and CRDF Calculations Corresponding to Figures S1 and S2 . . . . . S9

Table S3: Numerical Values of the RDF and CRDF Calculations Corresponding to Figures S3 and S4 . . . . . S10

Table S4: Average values of the volumes (V) of the simulation box, the simulation box volumes (V), the thermal-expansion coefficient (α_p), the isothermal compressibility (β_T), and the isothermal bulk modulus (K_T) S11

Table S5: Thermodynamic properties for PLLA systems with 0, 1, 2, 6, and 12 m-% of water S12

Figure S1: Radial Distribution Function Plots for H11-Os, O10-H5, O4-H5, O4-(H7-H9), Os-C6, and O4-C2

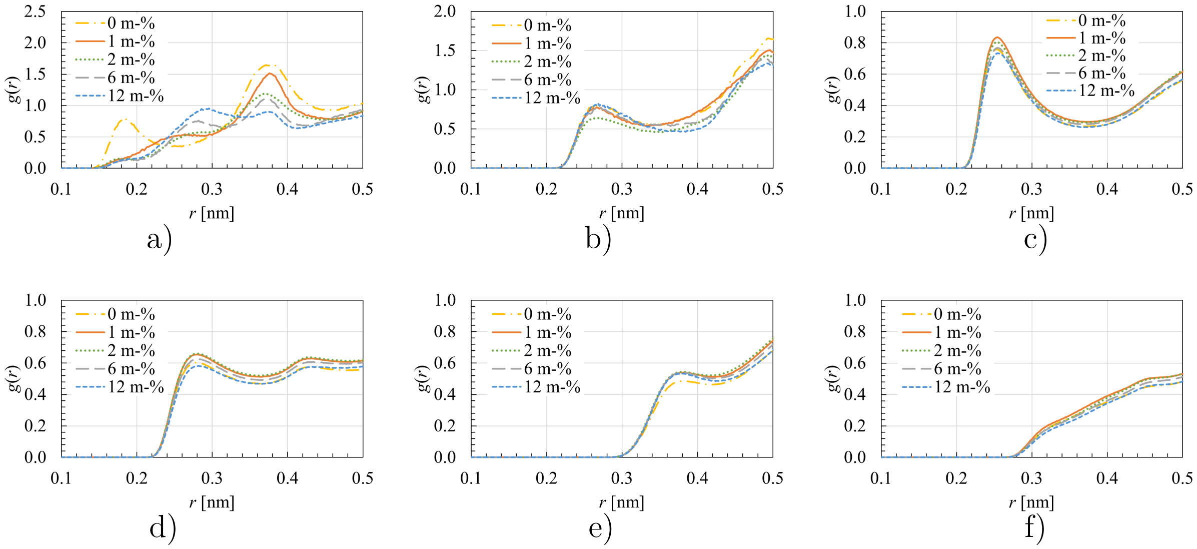

Figure S1: Plots of the RDFs; $g(r)$ as a function of the distance r [nm] between the atoms, for pairs of a) terminal alcohol hydrogen−ester oxygen (H11-Os), b) terminal alcohol oxygen−α-hydrogen (O10-H5), c) carbonyl oxygen−α-hydrogen (O4-H5), d) carbonyl oxygen−methyl hydrogens (O4-(H7-H9)), e) ester oxygen−methyl carbon (Os-C6), and f) carbonyl oxygen−carbonyl carbon (O4-C2), in pure PLLA (0 m-%, yellow $- \cdot -$) and with 1 (orange solid), 2 (green ··), 6 (gray −−), and 12 (blue short dash line) m-% of water in the PLLA matrix.

Figure S2: Cumulative Radial Distribution Function Plots for H11-Os, O10-H5, O4-H5, O4-(H7-H9), Os-C6, and O4-C2

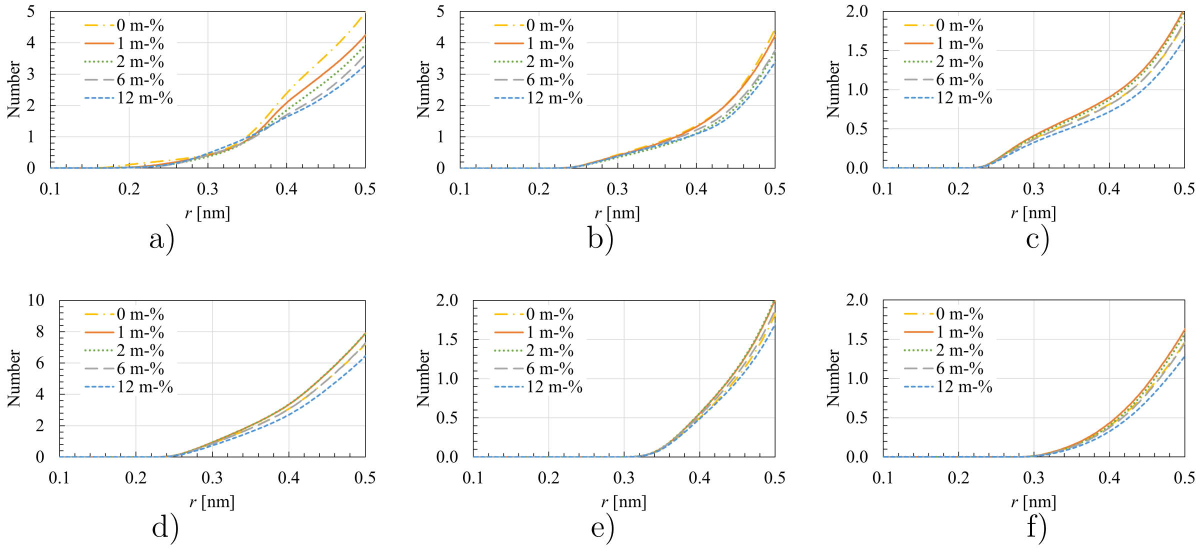

Figure S2: Plots of the CRDFs. Number (coordination number, coord#) as a function of the distance r [nm] between the atoms, for pairs of a) terminal alcohol hydrogen−ester oxygen (H11-Os), b) terminal alcohol oxygen−α-hydrogen (O10-H5), c) carbonyl oxygen−α-hydrogen (O4-H5), d) carbonyl oxygen−methyl hydrogens (O4-(H7-H9)), e) ester oxygen−methyl carbon (Os-C6), and f) carbonyl oxygen−carbonyl carbon (O4-C2), in pure PLLA (0 m-%, yellow − · −) and with 1 (orange solid), 2 (green ··), 6 (gray −−), and 12 (blue short dash line) m-% of water in the PLLA matrix.

Figures S3 and S4: Radial and Cumulative Radial Distribution Function Plots for Os-(H7-H9), Os-H5, and Os-C3

Ester oxygens (Os) can get closer to and coordinate with methyl hydrogens (H7-H9) only slightly better (Figure S3a and Figure S4a, Table S3) than methyl carbons (C6), because hydrogens stretch out from methyl carbons, for which they provide the shield. However, the values of $g(r)$ are small, although the coord# increases at low water concentration.

The RDFs and CRDFs for contacts between ester oxygens (O1 alias Os, ref.) and α-hydrogens (H5) of the neighboring PLLA chains are presented in Figure S3b and Figure S4b, and the numerical values in Table S3. The terminal carboxylic acid OH oxygen, which has the same atom type (O1) as the ester oxygen, has been excluded from the calculations. The most probable contact between α-hydrogens (H5) and ester oxygens (Os) (at the peak maximum) is at the interatomic distance of 0.29 nm but the probability and the coordination number are both low (ca. 0.2, Table S3). Water seems to slightly increase both the probability and the coordination number between these atoms. However, α-hydrogens (H5) are more prone

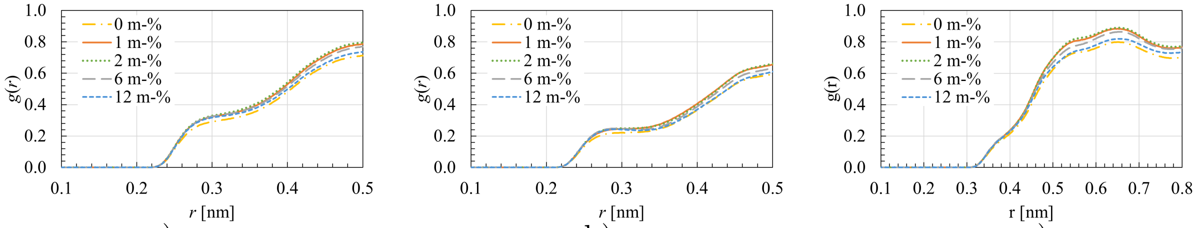

a) b) c)

Figure S3: Plots of the RDFs, $g(r)$ as a function of the distance r [nm] between the atoms, for pairs of a) ester oxygen−methyl hydrogens (Os-(H7-H9)), b) ester oxygen−α-hydrogen (Os-H5), and c) ester oxygen−α-carbon (Os-C3), in pure PLLA (0 m-%, yellow $- \cdot -$) and with 1 (orange solid), 2 (green ··), 6 (gray −−), and 12 (blue short dash line) m-% of water in the PLLA matrix.

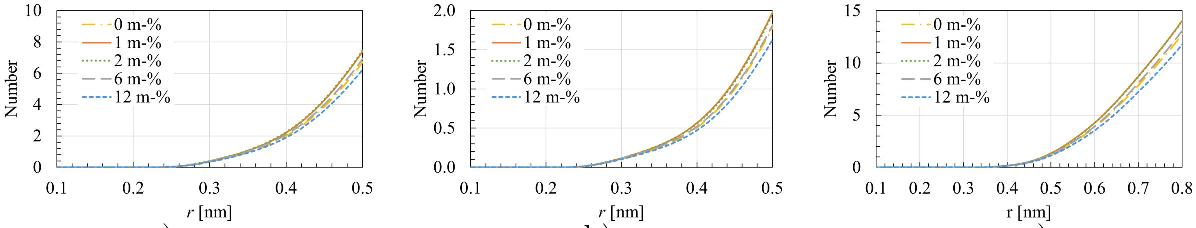

a) b) c)

Figure S4: Plots of the CRDFs. Number (coordination number, coord#) as a function of the distance r [nm] between the atoms, for pairs of a) ester oxygen−methyl hydrogens (Os-(H7-H9)), b) ester oxygen−α-hydrogen (Os-H5), and c) ester oxygen−α- carbon (Os-C3), in pure PLLA (0 m-%, yellow $- \cdot -$) and with 1 (orange solid), 2 (green ··), 6 (gray −−), and 12 (blue short dash line) m-% of water in the PLLA matrix.

to contact with chain end oxygens, O1 and O10, (Figures 3 and 4 (in the main text) and Figures S3b and S4b, respectively) and protruding carbonyl oxygens, O4, (Figures S1c and S2c) than with the ester oxygens of the polymer backbone.

The RDFs and CRDFs for contacts between ester oxygens (Os, ref) and α-carbons (C3) of the neighboring PLLA chains are presented in Figures S3c and S4c, respectively. The terminal OH oxygens of the carboxylic acid groups, which have the same atom type (O1) as the ester oxygen (O1 alias Os), have been excluded from the RDFs.

The RDF results indicate that the probability of contact between ester oxygens (Os) and α-carbons (C3) is very low (0.1-0.2) and the distance between the atoms is relatively long (ca. 0.37 nm, Table S3). Therefore, it can be concluded that direct contacts between pairs of C3-O1 atoms are possible but unlikely. The CRDFs (Figure S4c, Table S3) yield low coordination numbers of ca. 0.2 for all water contents. However, coordination increases slightly in the presence of water.

Figures S5 and S6: Volumes During the Equilibrations of the Replicates for Pure PLLA and the 2-m% System

All replicate systems were equilibrated for at least 3000 ns. The evolution of volume was examined to determine whether the systems had already reached equilibrium. Pure *a*-PLLA systems (0 m-% water) were chosen, as the volume had the largest fluctuation range in Table 1. The 2 m-% systems represent PLLA with water as the solvent. The 0 m-% and 2 m-% systems were additionally equilibrated to 4000 ns and 0 m-% S1 even further to 5000 ns to see if equilibrium had been reached for these systems.

All six systems in Figure S5 and S6 have plateaued at 3000 ns.

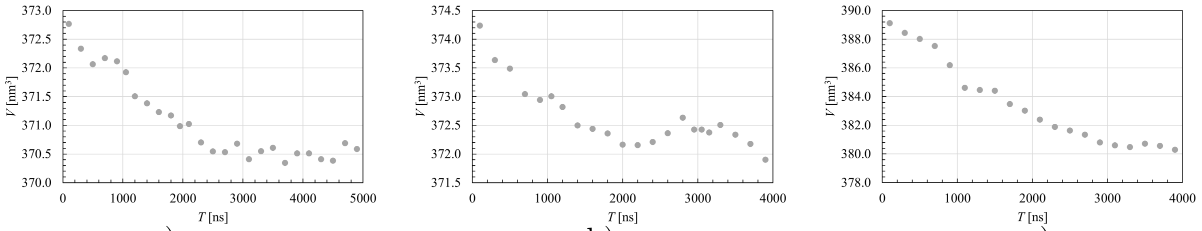

a) b) c)

Figure S5: Plots of the volumes in the three 0 m-% *a*-PLLA replicates during equilibration: a) S1, b) S2 and c) S3. The data points represent the average volume of 200 ns trajectory sections sampled during the equilibration of each system.

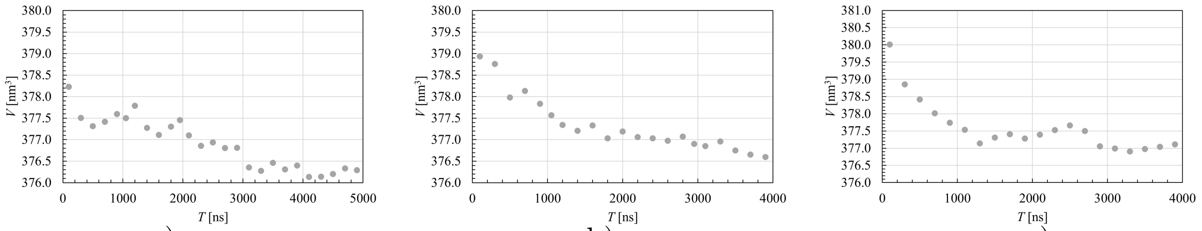

a) b) c)

Figure S6: Plots of the volumes in the three 2 m-% *a*-PLLA replicates during equilibration: a) S1, b) S2 and c) S3. The data points represent the average volume of 200 ns trajectory sections sampled during the equilibration of each system.

Figure S7: RDFs of the Three Independent Replicate Systems for the H11-O4 Atom Pair

The RDFs for the atom pairs are similar to each other when comparing the three independent replicate systems. For each m-% the probabilities may change between S1, S2 and S3 depending on how the molecules have settled in the systems. However, the distances of

the peaks are comparable in each set of three as seen, for example, in Figure S7. In higher concentrations where water molecules interfere more in the interactions of PLA, slight differences appear for the peak distances. All RDFs showed similar results between the atom pairs. The larges differences in peak placements appear in RDFs with a probability < 1. The systems have been equilibrated to a state stable enough to compare the results of the 15 systems. The molecules keep moving so a perfect equilibration is unlikely to happen but 3 μs is long enough to stabilize these systems. in the example Figure S7 water interacts with both atoms of the atom pair H11-O4. As more water is added to the systems more water molecules will push in between these two atoms decreasing the probability of interactions for PLA chains. There are 3750 O4-atoms in each system but only 25 H11-atoms.

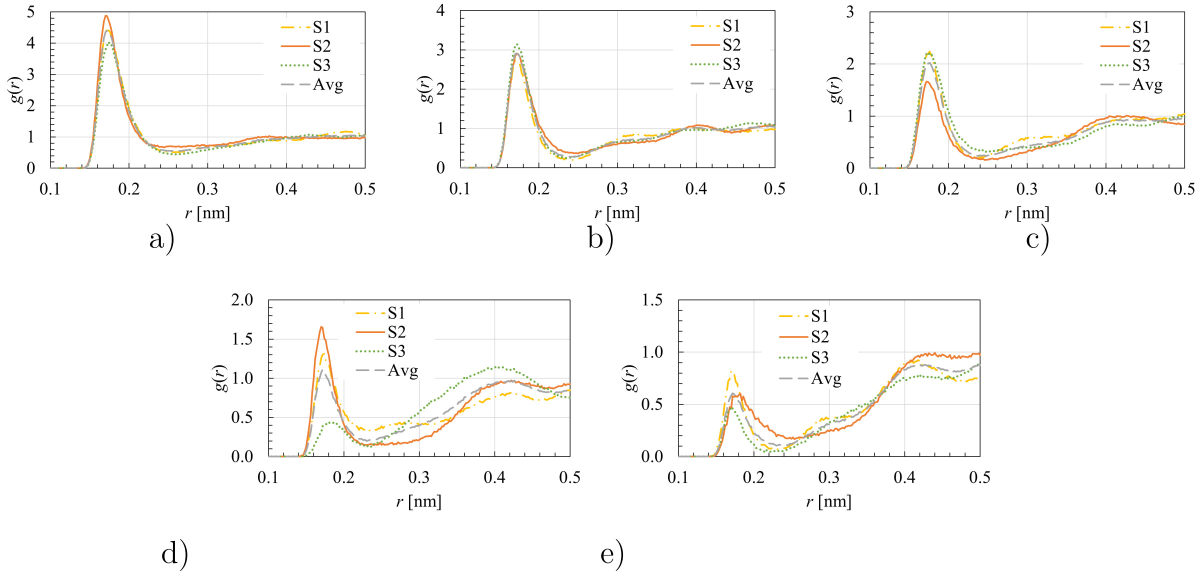

Figure S7: Plots of the RDFs: $g(r)$ as a function of the distance r [nm] between the atoms of the H11-O4 pairs, for a) 0, b) 1, c) 2, d) 6 and e) 12 m-%. S1, S2 and S3 are the three independent replicate systems and Avg is the average graph of these three systems also shown in Figure 3a.

Table S1: RDF peak maximum, $g(r_{\max})$, Standard error of the mean (SEM) of the RDF peak maximum, $\Delta g(r_{\max})$, separation distance at the first RDF peak maximum, $r_{\max}$ [nm], coordination number, coord#, SEM of the coord#, Δ coord#, and separation distance at the first RDF minimum, $r_{1st\,min}$ [nm], for the six atom-pair interactions that yield the highest g(r) values under the lowest-water-content conditions between adjacent PLLA chains; results for these interactions are also shown at other water contents, H_2O [m-%]. All values represent averages from three independent replicate systems.

H_2O [m-%]	$g(r_{\max})$	$\lvert\Delta g(r_{\max})\rvert$	$r_{\max}$ [nm]	coord#	$\lvert\Delta$ coord#$\rvert$	$r_{1st\,min}$ [nm]
			H11-O4			
0	4.40	0.27	0.17	0.86	0.03	0.25
1	2.91	0.13	0.17	0.47	0.03	0.24
2	2.02	0.20	0.18	0.35	0.04	0.24
6	1.11	0.37	0.17	0.18	0.04	0.23
12	0.61	0.09	0.17	0.09	0.02	0.23
			H10-O4			
0	3.67	0.15	0.18	0.94	0.02	0.28
1	3.15	0.25	0.18	0.92	0.05	0.28
2	3.20	0.40	0.18	0.77	0.03	0.28
6	2.17	0.22	0.18	0.49	0.04	0.26
12	2.01	0.39	0.18	0.39	0.04	0.26
			O1-C2			
0	1.84	0.03	0.39	3.16	0.06	0.45
1	1.77	0.04	0.39	3.16	0.05	0.45
2	1.75	0.07	0.39	2.97	0.11	0.45
6	1.64	0.08	0.39	2.59	0.03	0.45
12	1.64	0.15	0.40	2.49	0.15	0.45
			O10-C2			
0	1.81	0.18	0.38	2.87	0.12	0.45
1	1.34	0.13	0.39	2.26	0.05	0.45
2	1.01	0.08	0.39	1.22	0.07	0.42
6	0.91	0.07	0.39	1.48	0.06	0.44
12	0.70	0.04	0.39	0.96	0.04	0.43
			H10-Os			
0	0.41	0.08	0.20	0.10	0.01	0.23
1	0.90	0.26	0.20	0.42	0.07	0.28
2	0.67	0.10	0.20	0.10	0.05	0.23
6	0.52	0.08	0.24	0.30	0.03	0.29
12	0.50	0.16	0.20	0.05	0.02	0.21
			O1-H5			
0	0.73	0.12	0.27	0.75	0.05	0.34
1	0.89	0.04	0.28	0.79	0.04	0.34
2	0.98	0.04	0.27	0.85	0.02	0.34
6	0.93	0.05	0.27	1.09	0.01	0.37
12	0.82	0.16	0.27	0.70	0.09	0.35

Table S2: RDF peak maximum, $g(r_{\max})$, root mean square displacement (SEM) of the RDF peak maximum, $\Delta g(r_{\max})$, separation distance at the first RDF peak maximum, $r_{\max}$ [nm], coordination number, coord#, SEM of the coord#, Δ coord#, and separation distance at the first RDF minimum, $r_{1st\,min}$ [nm], for atom-pair interactions with the next six highest $g(r)$ values between adjacent PLLA chains at various water contents, H_2O [m-%]. All values represent averages from three independent replicate systems.

H_2O [m-%]	$g(r_{\max})$	$\lvert\Delta g(r_{\max})\rvert$	$r_{\max}$ [nm]	coord#	|Δ coord#|	$r_{1st\,min}$ [nm]
			H11-Os			
0	0.78	0.17	0.19	0.27	0.01	0.26
1	0.54	0.08	0.26	0.35	0.03	0.29
2	0.58	0.05	0.29	0.33	0.04	0.29
6	0.76	0.06	0.28	0.55	0.01*	0.32
12	0.95	0.07	0.30	0.85	0.02	0.34
			O10-H5			
0	0.80	0.08	0.26	0.78	0.04	0.35
1	0.77	0.05	0.27	0.65	0.03	0.33
2	0.65	0.05	0.27	0.56	0.03	0.33
6	0.77	0.02	0.27	0.71	0.02	0.35
12	0.83	0.08	0.27	0.80	0.11	0.36
			O4-H5			
0	0.76	0.07	0.25	0.68	0.07	0.37
1	0.84	0.01	0.25	0.75	0.01	0.37
2	0.81	0.01*	0.25	0.72	0.01*	0.37
6	0.77	0.01*	0.25	0.67	0.01*	0.37
12	0.73	0.02	0.25	0.60	0.02	0.37
			O4-(H7-H9)			
0	0.60	0.06	0.28	2.15	0.23	0.36
1	0.65	0.01	0.28	2.34	0.04	0.36
2	0.66	0.01*	0.28	2.34	0.01	0.36
6	0.63	0.01*	0.28	2.13	0.01*	0.36
12	0.58	0.03	0.28	1.87	0.09	0.36
			Os-C6			
0	0.49	0.05	0.38	0.65	0.07	0.42
1	0.54	0.01	0.38	0.77	0.01	0.42
2	0.54	0.01*	0.38	0.71	0.01*	0.41
6	0.54	0.01*	0.38	0.73	0.01*	0.42
12	0.53	0.03	0.38	0.71	0.04	0.42
			O4-C2			
0	0.45	0.05	0.46	1.30	0.14	0.49
1	0.51	0.01	0.46	1.44	0.02	0.49
2	0.50	0.01*	0.46	1.38	0.01*	0.49
6	0.48	0.01*	0.46	1.28	0.01*	0.49
12	0.46	0.02	0.46	1.13	0.04	0.49

*Rounded up to 0.01 from values in the range of 0.001 to 0.004

Table S3: RDF peak maximum, $g(r_{\mathrm{max}})$, Standard error of the mean (SEM) of the RDF peak maximum, $\Delta g(r_{\mathrm{max}})$, separation distance at the first RDF peak maximum, r_{max} [nm], coordination number, coord#, SEM of the coord#, Δ coord#, and separation distance at the first RDF minimum, $r_{1st\,min}$ [nm], for atom-pair interactions with the lowest three $g(r)$ values between adjacent PLLA chains at various water contents, H_2O [m-%]. All values represent averages from three independent replicate systems.

**H_2O [m-%]**	**$g(r_{\mathrm{max}})$**	**$\|\Delta g(r_{\mathrm{max}})\|$**	**r_{max} [nm]**	**coord#**	**|Δ coord#|**	**$r_{1st\,min}$ [nm]**
			Os-(H7-H9)			
0	0.24	0.02	0.27	0.58	0.06	0.32
1	0.26	0.01*	0.27	0.65	0.01	0.32
2	0.27	0.01*	0.27	0.65	0.01*	0.32
6	0.26	0.01*	0.27	0.62	0.01*	0.32
12	0.26	0.01	0.27	0.57	0.03	0.32
			Os-H5			
0	0.22	0.02	0.29	0.16	0.01	0.32
1	0.24	0.01*	0.29	0.18	0.01*	0.32
2	0.25	0.01*	0.29	0.18	0.01*	0.32
6	0.25	0.01*	0.29	0.17	0.01*	0.32
12	0.24	0.01	0.29	0.16	0.01	0.32
			Os-C3			
0	0.14	0.01	0.37	0.17	0.02	0.40
1	0.15	0.01*	0.37	0.18	0.01*	0.40
2	0.15	0.01*	0.37	0.18	0.01*	0.40
6	0.16	0.01*	0.37	0.17	0.01*	0.40
12	0.15	0.01	0.37	0.16	0.01	0.40

*Rounded up to 0.01 from values in the range of 0.001 to 0.004

Table S4: Average values of simulation box volumes (V), thermal-expansion coefficient (α_p), isothermal compressibility (β_T), and isothermal bulk modulus (K_T) for pure PLLA, PLLA–water systems with 1, 2, 6, and 12 m-% of water. Calculated values for the three independent replicate system groups (S1, S2, and S3).

H_2O [m-%]	V [nm^3]	$V \times 10^{-5}$ [m^3mol^{-1}]	$\alpha_p \times 10^{-4}$ [1/K]	$\beta_T \times 10^{-10}$ [Pa^{-1}]	$K_T \times 10^9$ [Pa]
**S1**					
0	370.6	5.96	4.05	4.27	2.34
1	373.5	6.00	3.89	4.24	2.36
2	376.7	6.05	3.87	4.13	2.42
6	392.2	6.30	4.56	4.17	2.40
12	418.6	6.72	5.45	4.17	2.40
**S2**					
0	372.4	5.98	4.03	4.49	2.23
1	373.2	5.99	3.99	4.31	2.32
2	376.9	6.05	3.98	4.13	2.42
6	391.0	6.28	4.41	4.09	2.44
12	418.2	6.72	5.21	4.13	2.42
**S3**					
0	380.7	6.11	3.78	4.53	2.21
1	373.4	6.00	4.01	4.33	2.31
2	377.0	6.05	3.95	4.36	2.29
6	392.8	6.31	4.26	3.95	2.53
12	417.6	6.70	5.79	4.27	2.34

Table S5: Thermodynamic properties for PLLA systems with 0, 1, 2, 6, and 12 m-% of water: enthalpy of vaporization (H), molar isobaric and specific heat capacity (C_p), and the difference $C_\mathrm{p} - C_\mathrm{V}$ between C_p and the heat capacity at constant volume (C_V). Calculated values for the three independent replicate system groups (S1, S2, and S3). Properties of pure water are given for reference.

H_2O [m-%]	H [kJ mol^{-1}]	H [kJ kg^{-1}]	C_p [J mol^{-1}K^{-1}]	C_p [J g^{-1}K^{-1}]	$C_p - C_V$ [J mol^{-1}K^{-1}]	$C_p - C_V$ $\times 10^{-3}$ [J g^{-1}K^{-1}]
**S1**						
0	123.2	1706	203	2.81	6.86	9.50
1	117.0	1685	198	2.82	6.17	8.80
2	110.9	1662	192	2.81	6.07	8.92
6	89.3	1567	180	2.96	7.43	12.23
12	68.3	1440	166	3.11	9.43	17.60
**S2**						
0	123.3	1708	201	2.79	6.50	9.00
1	116.9	1683	199	2.84	6.38	9.10
2	110.3	1654	192	2.82	6.45	9.47
6	89.2	1566	176	2.91	7.07	11.63
12	68.3	1439	166	3.11	8.69	16.21
**S3**						
0	123.9	1716	204	2.82	5.80	8.04
1	116.9	1685	198	2.83	6.40	9.14
2	110.9	1663	193	2.84	5.99	8.80
6	89.2	1565	179	2.94	6.88	11.31
12	68.3	1440	167	3.12	10.37	19.35
